# Efficacy and Safety of Luspatercept in the Treatment of β-Thalassemia: A Systematic Review

**DOI:** 10.7759/cureus.31570

**Published:** 2022-11-16

**Authors:** Ibrahim M Dighriri, Khawlah K Alrabghi, Dilveen M Sulaiman, Abdulrahman M Alruwaili, Nader S Alanazi, Al-maha A Al-Sadiq, ‌‏Amal M Hadadi, Bushra Y Sahli, Basil A Qasem, Manal T Alotaibi, Taif T Asiri, Salman M Majrashi, Noura T Alotibia, Afnan T Alhamyani, Amjad A Alharbi

**Affiliations:** 1 Department of Pharmacy, King Abdulaziz Specialist Hospital, Taif, SAU; 2 Department of Pharmacy, Al Qurayyat General Hospital, Al Qurayyat, SAU; 3 College of Pharmacy, University of Duhok, Duhok, IRQ; 4 Department of Pharmacy, Al Dawaa Medical Services Company, Sakaka, SAU; 5 Department of Pharmacy, King Salman Specialist Hospital, Hail, SAU; 6 Department of Pharmacy, Mouwasat Hospital, Jubail, SAU; 7 Department of Pharmacy, Community Pharmacy, Jazan, SAU; 8 College of Pharmacy, Jazan University, Jazan, SAU; 9 College of Pharmacy, Shaqra University, Al Dawadmi, SAU; 10 College of Pharmacy, King Khalid University, Abha, SAU; 11 College of Pharmacy, Prince Sattam Bin Abdulaziz University, Al Kharj, SAU; 12 College of Pharmacy, Taif University, Taif, SAU; 13 Pharmaceutical Care Services, King Salman Specialist Hospital, Hail Health Cluster, Ministry of Health, Hail, SAU

**Keywords:** reblozyl, safety, efficacy, β-thalassemia, luspatercept

## Abstract

β-thalassemia is characterized by the faulty generation of hemoglobin resulting in an elevated α/β globin ratio; this led to several patients needing red blood cell (RBC) transfusions for the rest of their lives. Luspatercept is an erythroid maturation test for treating various types of anemia, including β-thalassemia. It inhibits the Smad2/3 cascade and treats β-thalassemia by downregulating the transforming growth factor-beta (TGF-β) pathway. Luspatercept was evaluated in randomized controlled trials (RCTs). However, there is still limited data. Therefore, the study aims to review the current literature to assess the efficacy of luspatercept in cure β-thalassemia and its safety. From 2015 to 2022, searches were undertaken in PubMed, Google Scholar, and Cochrane. Only RCTs published in English were eligible for inclusion. The Cochrane Collaboration tool for bias assessment was used to analyze the quality of the publications. Our search strategy revealed 94 publications, of which 12 full-text papers were read and five were chosen for this review.All five trials included 1161 participants. Of whom, 153 (13.18%) entered phase 2, and 1008 (86.82%) entered phase 3. Two articles included 153 participants, of whom 70 (45.75%) were transfusion-dependent beta-thalassemia (TD) and 83 (54.25%) were non-transfusion-dependent beta-thalassemia (NTD) of phase 2. Three articles included 1008 participants, of whom 672 (66.67%) were given luspatercept and 336 (33.33%) were given a placebo. All participants in RCTs were 18 years of age or older. In phase 2, 0.2 to 1.25 mg/kg of luspatercept was given, and in phase 3, 1.0 to 1.25 mg/kg of luspatercept was given once every three weeks. In beta-thalassemia patients, luspatercept was more effective than a placebo and well tolerated. The high dose has shown promising results in the erythroid response, measured by a drop in blood transfusions or an average rise in hemoglobin levels. Luspatercept might make patients less likely to need RBC transfusions, improve their clinical results, and improve their quality of life. Adverse events were hyperuricemia, arthralgia, dizziness, influenza hypertension, and bone pain, but they were manageable.

## Introduction and background

Thalassemia is an inherited blood syndrome from the anemia family and causes a significant worldwide health burden [1]. Transfusions of red blood cells (RBCs) are a vital support therapy for patients suffering from anemia due to β-thalassemia [2]. An unbalanced ratio of α/β globin results from improperly produced globin chains in hemoglobin [3]. The degree of inefficient erythropoiesis and chronic anemia is determined by mutations and subsequent modifiers that affect this imbalance [3,4]. The incidence of beta-thalassemia is one case per 1000,000 children [5]. Asia, Africa, and the United States have a high frequency of thalassemia [6]. Medically, β-thalassemia is categorized as either transfusion-dependent or non-transfusion-dependent. Transfusion-dependent β-thalassemia manifests in children and requires frequent transfusions of RBC to keep appropriate blood levels [4]. β-thalassemia causes reduced hemoglobin synthesis, ultimately leading to severe persistent anemia. Until 2019, the only available therapy for β-thalassemia was blood transfusions, which were associated with life-threatening side effects such as iron toxicity and organ damage [6,7]. Therefore, there is a great need for alternative, effective, and safe therapies such as luspatercept (Reblozyl®).

Luspatercept considers recombinant protein that stimulates late-stage erythropoiesis by binding to transforming growth factor-beta (TGF-β) ligands [8]. It reduces Smad2/3 signaling, improves late-stage erythroid formation in bone marrow, and improves animal hematological parameters. In clinical trials of β-thalassemia use, luspatercept resulted in a persistent excess in hemoglobin and a decline in transfusions [9].

Luspatercept was approved via the Food and Drug Administration (FDA) on November 8, 2019 [10]. It was also authorized in 2020 via the European Medicines Agency [11]. Luspatercept was used to cure β-thalassemia and treat myelodysplastic syndrome [12]. For individuals with β-thalassemia, luspatercept administers at 1 mg/kg subcutaneously every three weeks [9]. Luspatercept shows a wide therapeutic margin in pharmacokinetics and response analyses. Although elevated serum exposure to luspatercept increased hemoglobin and was expected to increase efficacy [11]. In the phase 3 trial, luspatercept showed adverse events such as arthralgia, hyperuricemia, hypertension, and bone pain [13]. To prevent its toxicity, the dose should be regulated according to hemoglobin levels and should not exceed 1.25 mg/kg [10]. Luspatercept was evaluated in randomized controlled trials (RCTs). However, there is still limited data. As a result, this study intends to conduct a systematic review of the current literature to assess luspatercept efficacy and safety in β-thalassemia.

## Review

Methodology

Study Design

Preferred reporting items for systematic reviews and meta-analyses (PRISMA) protocol in 2020 and the Cochrane Handbook were followed for this study [14,15].

Research Question

Is the use of luspatercept in β-thalassemia efficacy and safe?

Sources of Data and Search Strategy

Search terms comprise database-specific indexed phrases and keywords to improve search efficiency. Searches were conducted in PubMed, Cochrane, and Google Scholar from 2015 to 2022. All the authors chose the studies. We review the remaining articles at the title and abstract level after eliminating duplicates and according to inclusion criteria. Then, the entire texts of the enduring records were gathered to see if they met all the requirements to be included.

Inclusion and Exclusion Criteria

RCTs that evaluated the efficacy of luspatercept in cure β-thalassemia and its safety were included. Excluded studies were preclinical, nonrandomized comparative, observational studies, reviews, dissertations, quasi-experimental research, and studies written in languages other than English. Only the most complete studies that looked at the same people were considered. The entire publication was included if studies were published in complete article form and as an abstract.

Selection of Studies and Data Extraction

EndNote was utilized for citation management and duplication elimination. Severally, two authors reviewed the titles and then the abstracts during the first screening. The entire text was reviewed at the second screening, and information extracted from the studies was transferred to standardized data recording forms. The number of participants, dose, and main findings were recorded.

Quality Assessment

The bias evaluation tool developed by the Cochrane Collaboration was utilized to measure the quality. It is divided into seven significant domains: random generation, allocation concealment, patient and staff blinding, result from evaluation blinding, preliminary findings, biased outcome reporting, and other biases.

Dissemination and Ethical Issues

This systematic investigation relied heavily on secondary data. Therefore, our local institutional review board (IRB) approval was unnecessary, and we waived it.

Result

**Figure 1:**
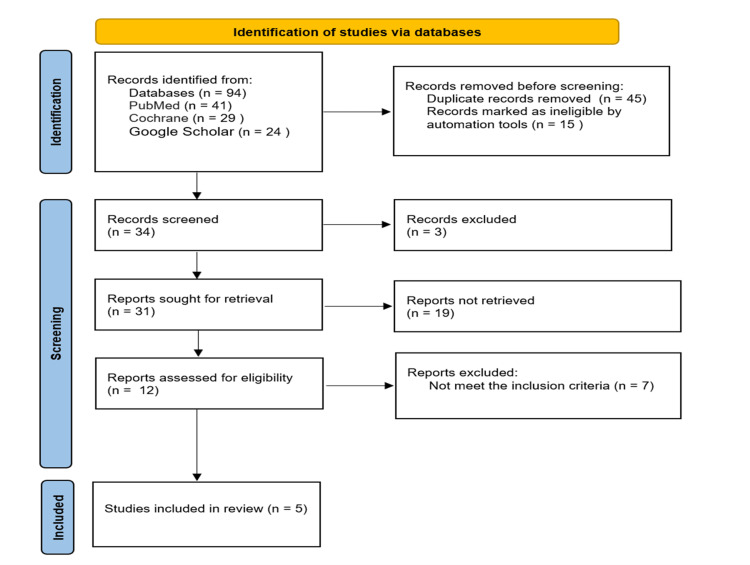
Diagram summarizing the study selection process using PRISMA. PRISMA: preferred reporting items for systematic reviews and meta-analyses.

We identified 94 articles using electronic databases like PubMed (41), Cochrane Library (29), and Google Scholar (24). After 60 articles were removed because 45 were duplicates and 15 were not relevant. Thirty-four were screened for titles and abstracts. A further 12 articles were assessed for eligibility after 22 were excluded because 17 studies were not RCTs and five studies did not measure efficacy or safety. Finally, five articles were included in this review (Figure 1).

**Figure 2:**
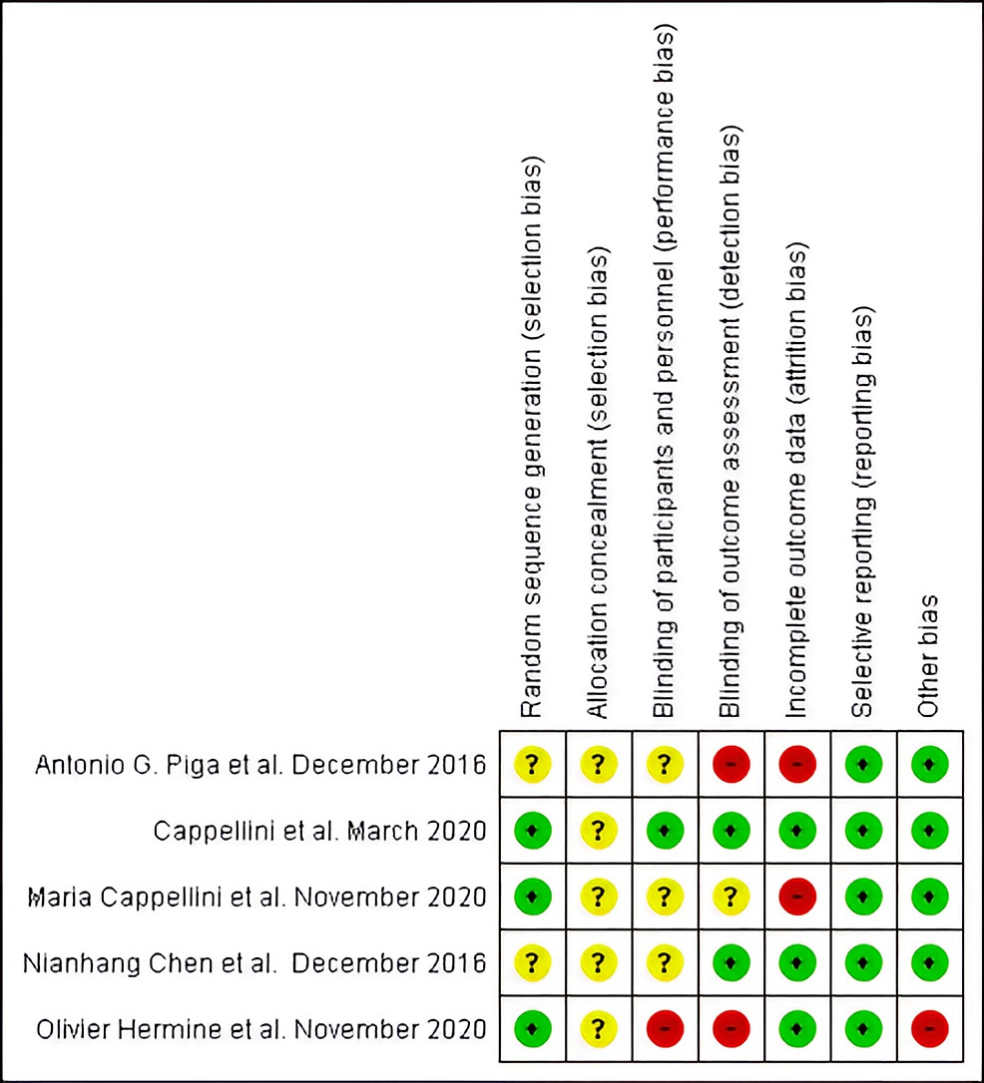
Assessment of the risk of bias green signifies a low bias, yellow signifies an unclear bias, and red signifies a high bias. Piga et al. [16]; Chen et al. [17]; Cappellini et al. [18]; Cappellini et al. [19]; and Hermine et al. [20]. Note: This image is the author's own creation.

The article's quality assessment showed the following: Three studies applied suitable randomization procedures, and the allocation concealment strategy needed to be clarified in all experiments. In one article, participants and personnel were blinded, and two RCTs had appropriate blinding of the outcome assessment. Due to a high dropout rate, two trials displayed a significant risk of bias according to insufficient outcome data; all RCTs had appropriate selective reporting. Four RCTs had a low risk of other biases (Figure 2).

A total of 1161 participants were included, of whom 153 (13.18%) entered phase 2 and 1008 (86.82%) entered phase 3. Two articles included 153 participants, of whom 70 (45.75%) were transfusion-dependent beta-thalassemia (TD) and 83 (54.25%) were non-transfusion-dependent beta-thalassemia (NTD) of phase 2. Three articles included 1008 participants, of whom 672 (66.67%) were given luspatercept and 336 (33.33%) were given a placebo. All participants in RCTs were 18 years of age or older. Luspatercept was given once every three weeks. Doses of luspatercept started from 0.2 to 1.25 mg/kg in phase 2 versus 1.0 mg per kg to 1.25 mg per kg in phase 3.

The phase 2 results revealed that the efficacy of luspatercept was demonstrated as the following median transfusion burden was eight units/12 weeks (range 4-15 units), and the mean and standard deviation (SD) of liver iron concentration (LIC) were 5.1 (5.3) mg/g Dw. Twenty (83%) and sixteen (67%) TD participants achieved a ≥33% and ≥50% diminished in transfusions over any twelve weeks, respectively. The median area under the curve (AUC) for NTD and TD responders was comparable (100 dg/mL versus 120 dg/mL). The frequency of responders for NTD and TD patients rose as AUC increased. According to population pharmacokinetics simulation, a starting dosage of 1.0 mg per kg luspatercept led to more than 90% of NTD patients and 50% of TD patients obtaining the median AUC concentration in responders. At the 0.8 mg/kg dosage, 50% of patients (NTD or TD) were expected to attain the desired AUC. Phase 2 results revealed that luspatercept was well tolerated. With greater AUC, grade 2-3 drug-related unfavorable events were more common (P<0.05). The adverse events were primarily mild to moderate. Musculoskeletal pain, myalgia, headaches, asthenia, and arthralgia were the most common side effects in TD people (Table 1).

Phase 3 results revealed that the efficacy of luspatercept following patients who have experienced a decrease in transfusion load of at least 33% from baseline between weeks thirteen through twenty-four, as well as a minimum decrease of two red-cell units over these 12 weeks, was significantly superior in the luspatercept versus the placebo (21.4% versus 4.5%, P<0.001). The number of participants who had a decrease in transfusion load of at minimum 33% over any twelve-week period was higher in the luspatercept versus the placebo (70.5% versus 29.5%), as was the percentage of those who experienced a reduction of at minimum 50% (40.2% versus 6.3%). The health-related quality of life assessed patients were (212 (94.6%) versus 104 (92.9%)) in the luspatercept versus in placebo, respectively. When assessing luspatercept responders, patients who administered luspatercept and had a 50% reduction in RBC transfusion load over twelve weeks were considerably more likely than those who administered placebo to have a clinically meaningful improvement in the physical component summary (31.1% versus 16.5%; P = 0.024) and physical functioning (30.0% versus 13.2%; P = 0.007) at week 48. Mean baseline serum ferritin, LIC, and myocardial T2* for luspatercept versus placebo arms were 2097 versus 1845 µg/L, 12.0 versus 10.1 mg/g Dw, and 33.5 versus 34.8 ms, respectively. Of 141 luspatercept-treated participants with baseline mean serum ferritin ≥1000 µg/L, 24 (17.0%) patients achieved post-baseline mean item Short Form Health Survey <1000 µg/L when assessed over weeks 1−24, versus 3 (5.0%) placebo-treated patients (Table 1).

**Table 1:** The characteristics of five included studies on luspatercept in β-thalassemia. TD: transfusion-dependent beta-thalassemia, NTD: non-transfusion-dependent beta-thalassemia, LIC: liver iron concentration, SD: standard deviation, AUC: area under the curve, RBC: red blood cell, RCTs: randomized controlled trials, Dw: dry weight.

Author name and publication date	Study design	Participants' numbers and age	Dose given	Main findings
Piga et al. 2016 [16]	RCT phase 2	Thirty TD patients were in the case study. Thirty-four NTD patients enrolled in the base study. ≥18 years old. Median age = 38.	Six cohorts (n = 35) were given doses ranging from 0.2 to 1.25 mg/kg. Cohort 29 and patients who moved to the extension trial (n = 51) were started on 0.8 mg/kg and gradually increased to 1.25 mg/kg.	In thirty TD participants, at baseline, the transfusion load was eight units/twelve weeks (range 4–15 units), and the mean (SD) of LIC = 5.1 (5.3) mg per g Dw. A total of twenty (83%) and sixteen (67%) TD participants achieved ≥33% and ≥50% diminished transfusion load over any twelve weeks compared to baseline, respectively. The response duration varied from twelve to forty-eight weeks. In thirty-four NTD patients, at baseline, hemoglobin was 8.7 g/dL (range 7.6–9.8 g/dL), and the LIC was 4.9 (3.4) mg/g Dw. Twenty-one (78%) and fifteen (56%) NTD patients accomplished ≥1.0 g/dL and ≥1.5 g/dL rises, respectively, in mean hemoglobin throughout the twelve weeks. Response duration varied and ranged from sixteen to more than seventy-two weeks, with no evidence of a decreasing Hgb response with time. Luspatercept was well tolerated. The adverse events were primarily mild to moderate. Musculoskeletal pain, myalgia, headaches, asthenia, and arthralgia were the most common side effects in people with TD. The efficacy of luspatercept was clinically significant in NTD patients (it raises hemoglobin and reduces LIC) and TD patients (diminishes RBC transfusions). The safety profile of luspatercept was favorable.
Chen et al. 2016 [17]	RCT phase 2	Several patients (n = 89). Of those, NTD patients were 49, and TD patients 40. ≥18 years old. Median age = 37.	Luspatercept was given once every three weeks. A dosage-finding phase (0.2 to 1.25 mg/kg) and an expansion cohort (0.8 to 1.25 mg/kg).	Higher luspatercept serum AUC in NTD patients was associated with a more significant rise in hemoglobin level (p<0.01). Most TD patients exhibited a diminished transfusion burden; no dose-dependent trend for a lowering in units transfused was seen, presumably because of the negligible exposure range studied. The AUC median for TD and NTD responders was comparable (100 dg/mL versus 120 dg/mL). The frequency dose as the AUC increased. According to population pharmacokinetics simulation, a starting dosage of 1.0 mg per kg luspatercept led to more than 90% of NTD patients and 50% of TD patients obtaining the median AUC concentration in responders. At the 0.8 mg/kg dosage, 50% of patients (NTD or TD) were expected to attain the desired AUC. With a greater AUC, grade 2-3 drug adverse reactions were more common (P<0.05). The most prevalent adverse events in NTD and TD patients were bone and muscular pain. However, these adverse events had no significant association with luspatercept exposure. Greater plasma luspatercept is associated with a heightened erythroid response and more common category two to three adverse events. According to exposure-response and pharmacokinetics simulation, a phase 3 starting dosage of 1.0 mg per kg and the intra-patient dose increased to 1.25 mg/kg based on patient response is supported.
Cappellini et al. 2020 [18]	RCT phase 3	Three hundred thirty-six patients were randomized to treatment. The luspatercept was 224 patients. The placebo was 112 patients. ≥18 years old. Median age = 30.	1.00 to 1.25 mg/kg	The participants who experienced a reduced transfusion load of at least thirty-three from baseline between weeks thirteen and twenty-four and a diminished of minimum two red-cell units over twelve weeks were significantly better on luspatercept compared to the placebo (21.4% versus 4.5%). The participants with a reduced transfusion load of at least 33% during the twelve weeks were higher in luspatercept than the placebo (70.5% versus 29.5%), as did those who experienced a reduction. With luspatercept, adverse effects: transitory bone pain, dizziness, high blood pressure, and hyperuricemia were more prevalent than with placebo. The patients with TD who reduced their transfusion load were much higher in luspatercept than the placebo, and only a few adverse events resulted in therapy termination.
Cappellini et al. 2020 [19]	RCT phase 3	Of three hundred thirty-six patients, 224 were given luspatercept and 112 to placebo. ≥18 years old.	Luspatercept starting dose: 1.0 mg/kg with titration up to 1.25 mg/kg	The health-related quality of life assessed patients were (212 (94.6%) versus 104 (92.9%)) in luspatercept versus in placebo, respectively. At week forty-eight, health-related quality of life questionnaire rates among patients remaining on therapy were more than 87.5% for both surveys. The mean scores on both primary and exploratory dimensions were steady throughout time in both treatment groups and did not vary at weeks twenty-four and forty-eight. When assessing luspatercept responders, patients who received luspatercept and had a 50% reduction in RBC transfusion load over twelve weeks were considerably more likely than those who received a placebo to achieve a clinically significant improvement in physical component summary (31.1% versus 16.5%; P = 0.024) and physical function (30.0% versus 13.2%; P = 0.007) at week forty-eight. Statistically significant differences among individuals accomplishing transfusion independence for any eight or twelve weeks for some 36-item short-form health survey domains, but no statistical difference was seen in patients achieving a ≥33% reduction in RBC transfusion load for quality of life domains. However, patients with improved scores were higher with luspatercept, especially at week forty-eight. Compared to placebo, patients with TD who responded to luspatercept were more likely to have clinically substantial improvements in health-related quality of life.
Hermine et al. 2020 [20]	RCT phase 3	Of three hundred thirty-six patients, 224 were given luspatercept and 112 to placebo. ≥ 18 years old.	Subcutaneously administer 1.0 to 1.25 mg per kg or placebo every three weeks for at least forty-eight weeks.	The mean baseline serum ferritin, LIC, and myocardial T2* of luspatercept compared to placebo were 2097 compared to 1845 µg/L, 12.0 compared to 10.1 mg per g Dw, and 33.5 compared to 34.8 ms, respectively. Of one hundred forty-one luspatercept participants with baseline serum ferritin ≥1000 µg/L, 24 (17.0%) patients achieved post-baseline mean item Short Form Health Survey <1000 µg/L when assessed over weeks 1−24 compared to 3 (5.0%) placebo-treated patients. During weeks 73–96, twenty-six (46.4%) luspatercept patients with mean serum ferritin ≥1000 µg/L achieved a baseline mean serum ferritin <1000 µg/L. At weeks twenty-four and forty-eight, 5 (4.2%) and 13 (9.7%) luspatercept patients, respectively, converted from LIC more than 3 mg/g Dw at baseline into ≤3 mg/g Dw, versus 4 of 61 (6.6%) and 4 (5.9%) placebo patients; 15 (14.3%) of luspatercept patients shifted from LIC >3 mg/g Dw at baseline to ≤3 mg/g Dw at week 96. About six (20.0%) patients with luspatercept changed from myocardial iron T2* ≤20 ms at the beginning to >20 ms a week forty-eight (versus 1/11 (9.1%) placebo patients); at week 96, six (25.0%) luspatercept patients shifted from ≤20 ms to >20 ms. During weeks 1–12, the deferasirox dose in luspatercept patients was 1477.08 mg and 1516.28 mg in placebo patients. During the first 48 weeks of treatment, the luspatercept and the placebo showed no discernible differences. However, the participants taking ≥1 iron chelation treatment gradually diminished in both responders. Compared to placebo-treated patients, a more significant percentage of luspatercept patients had reduced blood ferritin, LIC, and myocardial iron levels throughout the first forty-eight weeks, indicating a reduced hazard of iron overload. Lengthy luspatercept therapy resulted in a rising percentage of participants with ferritin levels of more than 1000 g/L and lower trends in total iron chelation drug usage.

**Figure 3:**
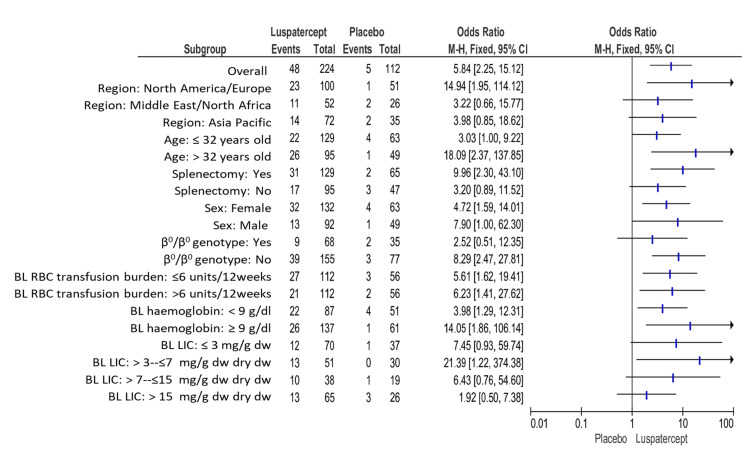
Transfusion load of at minimum 33% from baseline through weeks thirteen to twenty-four plus a reduction of at minimum two red-cell units throughout this twelve-week period. BL: baseline, CI: confidence interval, Dw: dry weight, LIC: liver iron concentration, RBC: red blood cell. Note: This image is the author's own creation.

The patients given luspatercept who experienced a decrease in transfusion load of at minimum 33% from baseline through weeks thirteen to twenty-four plus a reduction of at minimum two red-cell units throughout these twelve weeks that was substantially higher than in placebo (21.4% (48 of 224 patients) versus 4.5% (5 of 112); odds ratio, 5.84; 95% confidence interval (95% CI), 2.25 to 15.12) (Figure 3).

**Figure 4:**
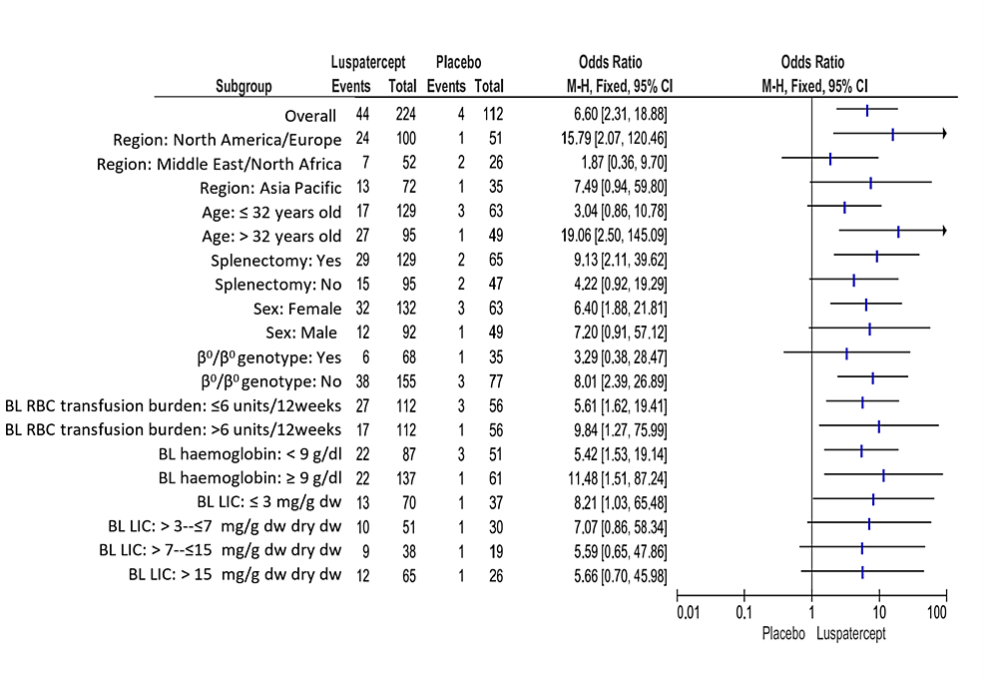
Transfusion load of at minimum 33% from baseline through weeks thirty-seven to forty-eight plus a reduction of at least two red-cell units throughout this twelve-week period. BL: baseline, CI: confidence interval, Dw: dry weight, LIC: liver iron concentration, RBC: red blood cell. Note: This image is the author's own creation.

The patients given luspatercept who experienced a decrease in transfusion load of at minimum 33% from baseline through weeks thirty-seven to forty-eight plus a diminish at least two red-cell units throughout these twelve weeks that was substantially higher than in placebo (19.6% (44 of 224 patients) versus 3.6% (4 of 112); odds ratio, 6.60; 95% CI, 2.31 to 18.88) (Figure 4).

**Figure 5:**
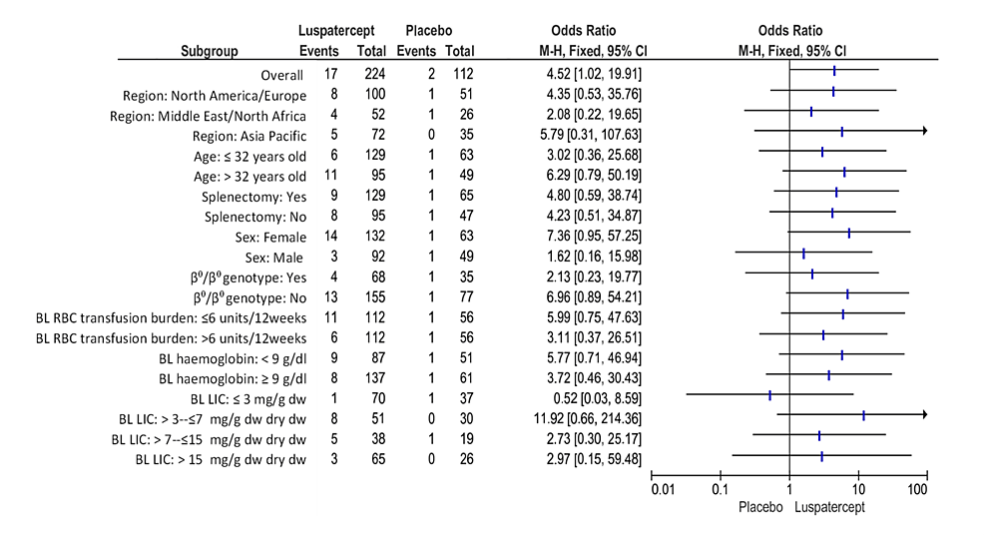
Transfusion load of at minimum 50% from baseline through weeks thirteen to forty-eight plus a reduction of at least two red-cell units throughout this twelve-week period. BL: baseline, CI: confidence interval, Dw: dry weight, LIC: liver iron concentration, RBC: red blood cell. Note: This image is the author's own creation.

The patients given luspatercept who experienced a decrease in transfusion load of at minimum 50% from baseline through weeks thirteen to forty-eight plus diminished at least two red-cell units throughout these twelve weeks that was substantially higher than in placebo (7.6% (17 of 224 patients) versus 1.8% (2 of 112); odds ratio, 4.52; 95% CI, 1.02 to 19.91) (Figure 5).

**Figure 6:**
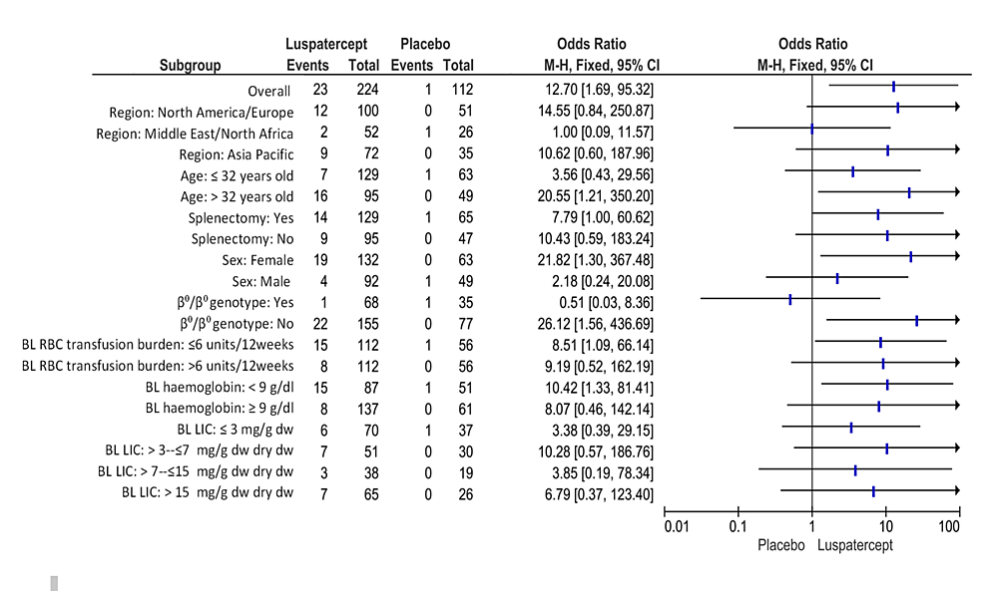
Transfusion load of at minimum 50% from baseline through weeks thirty-seven to forty-eight plus a reduction of at least two red-cell units throughout this twelve-week period. BL: baseline, CI: confidence interval, Dw: dry weight, LIC: liver iron concentration, RBC: red blood cell. Note: This image is the author's own creation.

The patients given luspatercept who experienced a decrease in transfusion load of at minimum 50% from baseline through weeks thirty-seven to forty-eight plus a diminish of at least two red-cell units throughout these twelve weeks that was substantially higher than in placebo (10.3% (23 of 224 patients) versus 0.9% (1 of 112); odds ratio, 12.70; 95% CI, 1.69 to 95.32) (Figure 6).

Discussion

This review's results revealed that using luspatercept for β-thalassemia is effective and safe. Also, more patients had a lower mean transfusion rate of at least 33% or 50% from baseline during the twelve-week or twenty-four-week interval. The most important aspects of treating people with thalassemia were blood transfusions and iron chelation medications [21]. Treatment goals include blood transfusion, hemoglobin increase, and quality of life enhancement [22]. The current treatment improves health and makes people live longer, but morbidity is still high [23].

This review found that the efficacy of luspatercept in phase 2 was clinically significant in both NTD and TD patients. Luspatercept was well tolerated, with no linked severe adverse events. With greater AUC, grade 2-3 drug-related adverse reactions were more common. A phase 1 trial demonstrated luspatercept activity in regular healthy volunteers [24]. Luspatercept safety was consistent with former experience and other patients [10]. In phase 3 of this study findings, long-term luspatercept therapy resulted in a rising percentage of participants with ferritin levels of more than 1000 g/L and lowering trends in total iron chelation therapy, indicating a reduced risk of iron overload complications. In practice, the red-cell units the patients receive per visit could be reduced, or the period's transfusion visits extended. Either can diminish the iron load, enhance patient adherence, and lower the associated disease burden [18]. Access to red-cell transfusion is challenging, especially in countries with limited resources where β-thalassemia is highly prevalent [1], consequently, unsatisfactory hemoglobin and delayed transfusion therapy commencement.

This review shows that, compared to a placebo, patients with TD-thalassemia who responded to luspatercept were more likely to have clinically substantial improvements in health-related quality of life. In contrast, luspatercept did not demonstrate an improved effect on the quality of life, but it was maintained in the population. This is because patients had a good quality of life at baseline, making a demonstration of the further improvement more challenging, or because effects on quality of life may not be observable in the initial phase of treatment but manifest only later [25]. The main treatments for anemia are transfusions and luspatercept [26]. Transfusion therapy alleviates anemia and inefficient erythropoiesis-related morbidities and symptoms. Inefficient erythropoiesis can impede development and growth and cause skeletal deformities, splenomegaly, and iron overload. Luspatercept can diminish transfusion required in beta-thalassemia patients [26].

Compared to placebo, luspatercept consistently performed better across all of the study's subgroups: age, origin, splenectomy, sex, pretransfusion hemoglobin, and iron level. These results are supported by preclinical data and an earlier open-label study including individuals with beta-thalassemia whose hemoglobin levels improved significantly [27]. Additionally, luspatercept has the potential to reduce transfusion reactions and iron input [26]. Longer-term follow-ups of patients administered luspatercept revealed that approximately 3% developed extra-medullary hematopoietic masses. These masses have sometimes caused severe complications, such as spinal cord compression [28]. The revised labeling for luspatercept states that patients should be monitored for extra-medullary masses, and luspatercept should be discontinued if these masses cause serious complications or cannot be controlled [29]. Serum ferritin levels were most affected by the decrease in transfusions, although the effect on iron index levels was less pronounced. Damage to the heart, liver and endocrine organs due to secondary iron excess is a severe problem with frequent red-cell transfusion treatment [21]. Considering the iron chelation treatment administered, it is essential to analyze these results carefully. Despite advancements in iron chelation therapy, there are obstacles to its efficacy and safety, and treatment access and adherence are not regular [3]. Lightening the load of transfusions should diminish iron consumption and, thus, the need for iron chelation therapy.

Luspatercept adverse reactions included hyperuricemia, bone pain, dizziness, cough, influenza, arthralgia, and hypertension. These adverse events were low-grade and limited, in line with the findings in a past study [30]. Past studies reported luspatercept known risk of thromboembolic events (TEEs) in people with splenectomy [31]. TEEs occurred in individuals with a history of splenectomy and other thromboembolic risk factors [32]. TEEs included deep vein thrombosis (DVT), pulmonary emboli, and ischemic stroke. Therefore, the benefit of luspatercept should be compared to the possible harm of TEEs and the use of thromboprophylaxis, according to clinical guidelines [32]. To prevent toxicity, dosing should not exceed 1.25 mg/kg in β-thalassemia and 1.75 mg/kg in myelodysplastic syndromes [33]. Luspatercept is stopped if the patient's transfusion demand does not decrease despite the maximum dose for nine weeks, if toxicities are unacceptable, or if extra-medullary masses develop [25].

## Conclusions

Luspatercept is considered effective and safe. It promotes late-stage RBC maturation and reduces the transfusion burden. Luspatercept improves long-term clinical outcomes as well as health-related quality of life in β-thalassemia patients. Furthermore, luspatercept reduced the mean transfusion rate by at least 33% or 50% from baseline over a twelve-week or twenty-four-week period. Luspatercept consistently outperformed placebo in age, origin, splenectomy, sex, pretransfusion hemoglobin, and iron level. Adverse events were bone pain, cough, hypertension, dizziness, and hyperuricemia, which were manageable. Higher plasma luspatercept is linked to increased erythroid response and is a more prevalent unfavorable event. To avoid toxicity, the dose in β-thalassemia should not exceed 1.25 mg/kg.
